# Digital knowledge translation tools for sexual and reproductive health information to adolescents: an evidence gap-map

**DOI:** 10.1177/26334941241307881

**Published:** 2024-12-18

**Authors:** Salima Meherali, Amber Hussain, Komal Abdul Rahim, Sobia Idrees, Soumyadeep Bhaumik, Megan Kennedy, Zohra S. Lassi

**Affiliations:** University of Alberta, Edmonton, AB, Canada; Edmonton Clinic Health, Faculty of Nursing, University of Alberta,11405 87 Ave NW, Edmonton, Alberta, Canada T6G 1C9; Aga Khan University, Karachi, Sindh, Pakistan; University of Alberta, Edmonton, AB, Canada; The George Institute for Global Health, Newtown, NSW, Australia; University of Alberta, Edmonton, AB, Canada; University of Adelaide, Adelaide, SA, Australia

**Keywords:** adolescents, sexual and reproductive health, digital tools, knowledge translation, evidence gap-map

## Abstract

**Background:**

Digital knowledge translation (KT) interventions play a crucial role in advancing adolescent sexual and reproductive health (ASRH). Despite the extensive literature on their effectiveness, there’s a lack of synthesized evidence on the efficacy of digital KT tools for adolescent ASRH globally.

**Objectives:**

This review aimed to systematically identify and map existing empirical evidence on digital KT tools targeting ASRH outcomes and identify research gaps.

**Design:**

The review employed an evidence gap-map (EGM) approach following 2020 PRISMA reporting guidelines.

**Data sources and methods:**

A comprehensive literature search was conducted across databases including Medline, EMBASE, Global Health, CINAHL, Scopus, and Cochrane. Covidence software was used for data management. EPPI-Mapper software was used to synthesize findings and develop a graphical EGM.

**Results:**

The EGM comprises 68 studies: 59 experimental and 9 systematic reviews, predominantly from African (19 studies) and American regions (22 studies), with limited research from the Eastern Mediterranean and South East Asian regions. It examines digital KT tools’ influence on sexual and reproductive health (SRH) outcomes, identifying research gaps. Websites are extensively studied for their impact on adolescent behavior, knowledge, attitude, and self-efficacy, yet research on their effects on ASRH and health services access is limited. Similarly, mobile apps and short message service (SMS)/text messages impact various aspects of SRH outcomes, but research on their effects on health services utilization is insufficient. Interventions like digital pamphlets and gaming lack exploration in health service access. OTT media and social media need further investigation. Mass media, including radio, television, and podcasts, are largely unexplored in adolescent SRH outcomes. Topics such as menstrual hygiene, abortion, and sexual and intimate partner violence also lack research.

**Conclusion:**

The review underscores the dominance of certain KT tool interventions like SMS and websites. Despite advancements, research gaps persist in exploring diverse digital platforms on underrepresented outcomes globally. Future research should expand exploration across digital platforms and broaden the scope of outcome measures.

**Trial registration:**

The protocol is registered with PROSPERO (CRD42022373970).

## Introduction

According to the World Health Organization (WHO) 2023, adolescence is a crucial phase typically ranging from aged 10 to 19 years, marked by significant physical, emotional, cognitive, and social changes.^1^ Adolescents experience rapid changes in their physical and psychosocial development including pubertal changes and increased demand for independence, self-discovery, and the formation of one’s identity.^1,2^ Adolescents are more prone to acquiring sexually transmitted infections (STIs) and facing unexpected pregnancies, as a result of various factors, such as behavioral and social aspects.^1,3^ According to the Centre for Disease Control and Prevention (2021), 26 million new cases of STIs were reported in the year 2018 and almost half of the new STIs cases were among youth (15–24 years).^4^ The most common contributing factor to these infections is limited knowledge about sexual health, including how STIs are transmitted and prevented.^5^ In addition, adolescents may face barriers in accessing sexual health services, and contraceptive use, including concerns about confidentiality, stigma, lack of awareness about available services, and judgmental attitudes of healthcare professionals.^5,6^ As a result, they engage in risky sexual behaviors, such as unprotected sex or having multiple sexual partners, which increases their vulnerability to STIs.^6,7^

Adolescents’ sexual and reproductive health (ASRH) needs and issues are a critical aspect of their overall health and well-being. Evidence reports that adolescents are not equipped with specific sexual and reproductive health (SRH) education.^7,8^ Therefore, it is imperative to utilize evidence-based innovative and novel approaches to address educational needs among this population. Among the others, comprehensive sexuality education and access to reproductive health services remain a priority for healthy sexual development.^1,9^ Open and non-judgmental communication, along with the provision of accurate information and accessible services, plays a vital role in supporting adolescents in making informed decisions about their sexual health. Bridging the gap between research and practice ensures that valuable insights and evidence contribute to informed decision-making, policy development, and improvements in professional practices—a process called knowledge translation (KT).^10^

KT is particularly crucial in fields where evidence-based decision-making is essential. This process involves developing and employing strategies and tools to integrate research findings into practice.^10^ There are several digital KT tools such as mobile phones, websites, mobile apps, short message service (SMS)/text messages, YouTube, Facebook, Twitter, Instagram, WeChat, and other social media platforms that have been identified as useful public health tools, particularly to promote SRH among adolescents.^8,9,11,12^ Several studies reported the positive use of digital tools in addressing SRH for adolescents; these include: maintaining privacy, anonymity,^12–14^ and convenience,^15^ making it a valuable way to provide accurate information about sexual health to adolescents.^6,8,9,16^ Other studies reported text messaging through mobile phone technology to increase awareness of adolescents to prevent STIs/HIV and improve safe sexual practices,^17–19^ and chatbots—a user-friendly digital tool for adolescents to maintain confidentiality of their queries related to topics around sex and sexual activities.^20^

Although there is sufficient evidence available on the effectiveness of digital tools in addressing adolescent SRH, there is a lack of synthesis of literature on the available digital or mHealth KT tools. Also, there is a lack of evidence on which digital KT tools prove to be more reliable and effective sources of SRH information for adolescents. Therefore, this review aimed to synthesize current and available evidence on the usefulness of digital KT tools to improve ASRH.

*Study aims and objectives*: This review aimed to identify, map, and describe existing empirical evidence on the digital KT tools designed to enhance awareness of SRH among adolescents globally. The specific objectives of this evidence gap-map (EGM) are to:

1. identify, assess, and report on empirical studies that describe the development, implementation, and/or evaluation of adolescent SRH digital KT tools;
2. identify current uses, purposes, and methods in the development of digital KT tools;
3. describe the characteristics of digital KT tools studies: such as target population, sample size, age of the participants, sex/gender, and regions
4. identify research gaps in the literature

## Methods

The study is conducted in alignment with standard methodologies for the development of EGM as detailed in our previously published protocol.^21^ An EGM is an emerging process that presents visual representations that highlight the existing evidence on a specific subject, illustrating where research has been conducted and where there are gaps in knowledge.^21^ This review followed a priori-developed^22^ and is registered with PROSPERO (CRD42022373970). The PRISMA (Preferred Reporting Items for Systematic Reviews and Meta-Analyses) guidelines have been followed to ensure comprehensive and transparent reporting of the research process.^23^ We followed the below-mentioned specific eligibility criteria to include studies in the EGM.

- *Topics of interest*: We included studies reported digital KT tools on SRH topics such as knowledge, attitude, and efficacy of SRH, pregnancy and birth, abortion, HIV, AIDS, and other STIs testing and their incidence, sexual and intimate partner violence, sexual behavior, menstrual hygiene, family planning and contraception use, communication and support related to SRH, and access to SRH services.
- *Population*: We included studies conducted on adolescents aged between 10 and 19 years. We also included studies if they included mixed population inclusive of adolescents age group.
- *Exposure/Intervention*: We included studies assessing digital KT tools for disseminating SRH information to adolescents. These KT tools included websites, mobile apps, SMS/text messages, digital pamphlets, brochures, digital storytelling and gaming, podcasts, mass media such as radio messages or videos on television, social media such as Instagram TikTok, Facebook, Twitter, YouTube, and OTT platforms such as Netflix, Prime, YouTube, online films, and videos.
- *Comparison*: We included studies comparing the above-mentioned KT tools with no interventions, the standard of care, or other interventions such as self-directed learning, traditional teaching (lecture), reminders, educational website, and controlled messages.
- *Setting*: We included studies conducted globally regardless of the settings (healthcare organizations, community, educational setting, etc.) or context of its conduct.
- *Timeframe and language*: Studies and reports published from 2010 onward were included in the review to capture current advancements and practices in the field. Due to language limitations of researchers, literature published only in the English language was considered to be included in the review.
- *Types of studies*: We included experimental studies (randomized/cluster randomized and non-randomized controlled trials, including quasi-randomized, controlled before-after, and interrupted time series), and observational studies, that is, including prospective cohort and case-control studies. The experimental and observational studies are only included in the presence of control/comparison arms. Studies with a historical control arm were excluded to focus on contemporary practices. Studies such as cross-sectional studies, case reports, series, editorials, and commentaries were excluded from this review. We included systematic reviews; however, following consistent criteria with the primary studies, systematic reviews with no comparison arm were excluded from this review. None of the scoping reviews was found relevant to be included in this review. Other types of reviews such as narrative reviews were excluded based on inconsistent and incomprehensive search strategies used in those reviews and lack of methodological robustness.

The search strategy for this review is reported in adherence to the PRISMA for Searching (PRISMA-S) extension^24^ (Appendix A). The search strategy was developed by an experienced health sciences librarian at the University of Alberta (MK) in consultation with the research team. The following databases were searched individually from inception to present: Medline (1946–present), EMBASE (1974–present), and Global Health (1910–present) via OVID; Cumulative Index to Nursing and Allied Health Literature (CINAHL, 1936–present) via EBSCOhost; Scopus (1976–present); and Cochrane Library (1993–present) via Wiley. The search strategy was derived from four main concepts: (1) adolescents, teenagers, or young adults; (2) SRH or health services including vocabulary related to contraception, family planning, pregnancy, STIs, and gender-based violence; (3) digital communication tools such as websites, online messaging, smartphones, mobile applications, social media, podcasts, television, or digital information; (4) KT including vocabularies such as information dissemination, research innovation, knowledge transfer, implementation science, research into practice, knowledge into practice, and evidence-based practice. Bibliographic databases were searched using a combination of natural language (keywords) and subject headings, such as Medical Subject Headings (MeSH), wherever they are available. Items such as books, book chapters, editorials, conference materials, and opinion pieces were removed from the results and a publication date limit of 2010–present was applied. A preliminary search for OVID Medline was developed and executed in October 2022 to determine the feasibility of this project and test the scope. An updated search was completed in October 2023 for this review. Covidence web-based software was used for the deduplication of database search results and for facilitating the title/abstract screening and full-text screening phases.

All the studies identified from the databases were imported to Covidence (an online screening software), and two independent reviewers completed the first level (title/abstract) (SI, KR) and second-level of screening (full-text) (AH, KR). Disagreements were resolved by consensus among the two reviewers. The reference list of all the included studies was scanned and searched to include any relevant study that may have been missed during searching of databases. For data extraction, we used a standardized data extraction form to extract descriptive data from all studies meeting our inclusion criteria. Data extracted from each study include bibliographic details, KT tool types and descriptions, outcome types and descriptions, study design, context/geographical information, and details on the outcome and quality of the included studies. Two review authors independently extracted the data (SI, AH), and discrepancies were resolved through discussion until consensus was achieved or by consulting a third reviewer (SM) if required. The PRISMA chart was used to document inclusion and exclusion decisions and ensure transparency and rigor in the reporting of the studies (Appendix A).

Data from the review is visually synthesized in an EGM using EPPI-Mapper, a tool developed by the EPPI-Centre at University College London (UCL). The 2D graphical EGM is presented with an accompanying narrative. Rows of the EGM list digital KT strategies and columns components of outcomes and other relevant data coding. Each cell shows the number and quality of evidence for digital KT strategies. We conducted the quality assessment of the included studies using tool ROB2 developed by the Cochrane Collaboration, for randomized control trials (RCTs), ROB1 for quasi-experimental studies, and AMSTAR2 for systematic reviews.^25,26^ The EGM identified areas with high-quality, evidence-based digital KT tools and areas where few or no KT tools exist (for targeted KT tool development and research/policy prioritization).

## Results

We identified 18,060 studies from electronic databases and finally included 68 studies in the EGM. The PRISMA study flow chart for the study is shown in Appendix A. Out of the 68 included studies, 59 were primary effectiveness studies and 9 were systematic reviews. The majority of primary studies conducted were RCTs (48 studies).^27–74^ Four studies were clustered RCTs,^75–78^ seven were quasi-experimental studies,^79–85^ and nine were review articles.^7–9,12,14,16,17,20,86^

The majority of the evidence comes from African (19 studies),^31,35,36,42–44,48,56,57,61,62,69,70,72,75,80–82,84^ and American regions (22 studies).^27,29,30,32–34,38,40,45–47,49,50,53,58–60,63–65,74,77^ Eight studies were based in European^28,37,39,41,51,55,66,76^ and eight in Western pacific region,^54,67,68,71,73,78,79,83^ while only one from Eastern Mediterriean^52^ and one from South East Asian region.^85^

**Figure 1.**
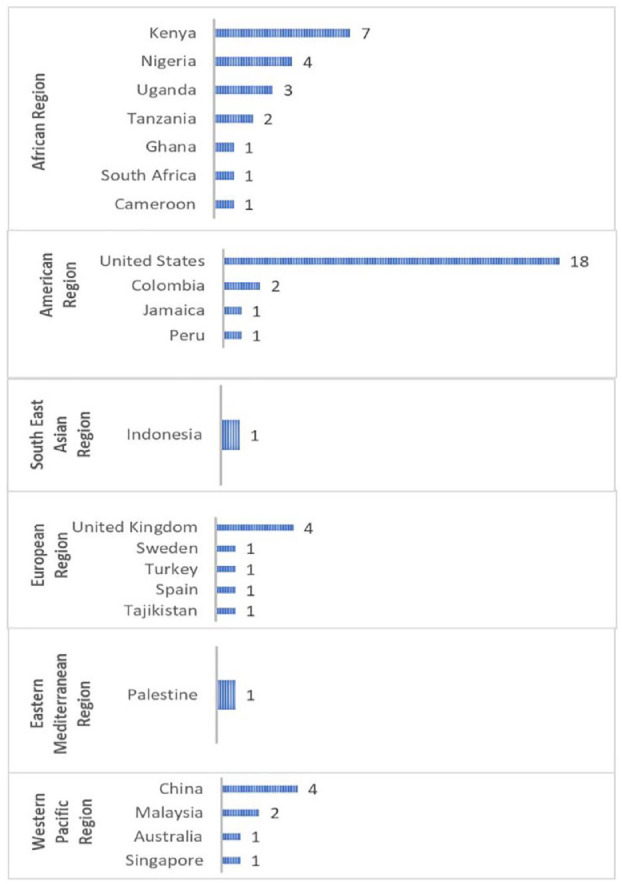
Evidence by region and country.

The evidence base from 59 studies (excluding review articles) was concentrated in 23 countries. The highest evidence comes from African and American regions, with the United States having the highest evidence among other countries (18 studies),^27,29,32,33,38,40,45,47,49,50,53,59,60,63–65,74,77^, followed by Kenya with 7 studies.^42,43,48,56,62,69,84^ European region displays a varied range of evidence among countries, with the United Kingdom having the highest (four studies),^28,37,66,76^ while Western pacific region showcases a mix of low-to-moderate counts across its listed countries, with China having the most evidence (four studies; Figure 1).^68,71,73,78^ Among nine review studies, six studies focused on a global perspective,^9,12,14,16,20,86^ while two targeted low and middle-income countries,^7,17^ and one specifically centered on Sub-Saharan Africa^8^.

This EGM is comprised of data from 43,382 participants in 59 primary studies. Sample sizes ranged from 50 to 8999 participants.^29,33^ Of the 59 primary studies, 22 studies had only female participants,^29,31,39–41,43,46,47,49,52,59–61,65,66,70,71,74,75,77,78,84^ 2 studies only had male participants,^38,56^ 5 studies had males who had sex with male participants (MSM),^34,45,53,63,67^ and the remaining 30 studies had both male and female participants^27,28,30,32,33,35–37,42,44,48,50,51,54,55,57,58,62,64,68,69,72,73,76,79–83,85^ (refer to Appendices B and C).

**Figure 2.**
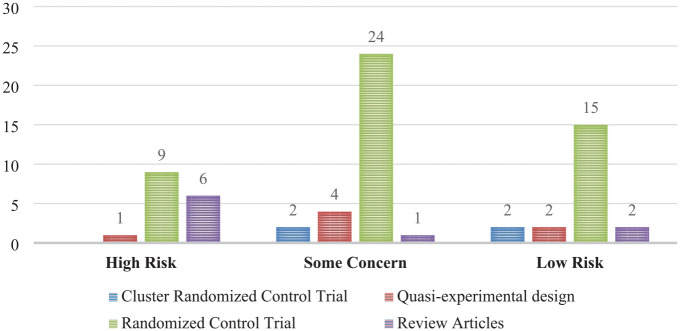
Quality appraisal of studies.

In terms of quality appraisal, the majority of studies included were rated moderate quality with some concerns (31 studies)^20,28,30,34,38,39,41–45,47,49,50,53,58,62–64,67,69–73,75,77,79,80,82,85^ largely due to inadequate reporting of methodological details. Sixteen studies had low quality with high risks,^7,9,12,14,17,27,32,33,35,48,59,65,68,74,81,86^ and 21 were of good quality that had a low risk^8,16,29,31,36,37,40,46,51,52,54–57,60,61,66,76,78,83,84^ (Figure 2).

A 2D graphical EGM was developed on EPPI-Mapper with a bubble map view consisting of rows and columns. Rows were types of KT tools divided into broad categories like website, mobile app, SMS/text messages, digital technology (pamphlet/brochure/storytelling/gaming), OTT media (Netflix, Prime, YouTube, online film, video), social media (Facebook, Instagram, WhatsApp, TikTok, Snapchat, LinkedIn), mass media (radio and television), and podcast. The columns encompass outcome categories grouped into broader categories, each with subcategories such as adolescent behaviors (sexual behavior, menstrual hygiene, contraception and prevention, communication, and support-seeking), adolescent knowledge, attitude and empowerment (knowledge and awareness, attitudes, self-efficacy), adolescent SRH outcomes (pregnancy and birth, abortion, HIV testing and incidence, sexual and intimate partner violence), and health services (accessing and utilizing services). The quality appraisals are highlighted by color codes: green for low risk of bias/high-quality, yellow for some concerns of bias/moderate quality, and red for high risk of bias/low-quality assessments. More detailed and dynamic versions that enable filtering by each specific subgroup with linked study references and additional study characteristics are available at the link here OR


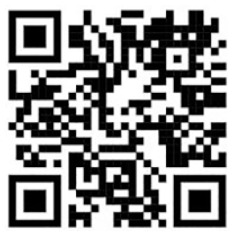


The EGM examined how different digital mediums affect adolescent SRH outcomes based on available studies. While most of the studies examined individual digital KT tools separately within a specific study, few studies explored the combined effects of multiple tools in a single study. Similarly, in some instances, certain studies reported more than one piece of SRH information. Websites wield significant influence over adolescent behavior, as evidenced by 12 studies,^8,16,17,30,39,45,50,65,66,71,74,81^ while impacting knowledge, attitude, and self-efficacy in 16 studies.^8,16,17,30,33,39,50,53,54,65,66,71,74,78,81,83^ However, only four studies have delved into their effects on adolescent SRH outcomes^16,50,54,67^ and merely two have investigated their impact on health services access and utilization.^53,54^ Mobile apps have exhibited impacts on adolescent behavior (14 studies),^7,17,32,40,49,51,55,62,63,68,69,75,77,82^ knowledge, attitude and self-efficacy (13 studies),^7,17,32,40,48,49,51,62,68,69,77,82,85^ adolescent SRH outcomes (4 studies),^32,55,63,82^ and healthcare access and utilization (only 1 study).^7^ SMS/text messages have emerged as highly influential, affecting adolescent behavior (22 studies),^7–9,12,17,20,27–29,37,38,42,43,49,51,52,56,60,70,72,84,86^ knowledge, attitude and self-efficacy (16 studies),^7,8,12,17,38,42,49,51,52,58,60,61,70,73,79,86^ SRH outcomes (13 studies),^9,12,28,31,37,38,42,52,60,61,72,84,86^ and to some extent, health services access and utilization (4 studies).^7,29,38,52^ Interventions involving digital technology like digital pamphlets, brochures, storytelling, and gaming have limited research but exhibit an impact on behavior (3 studies),^64,80,81^ and knowledge and attitude (6 studies),^35,44,57,64,80,81^ necessitating further exploration in terms of adolescent SRH outcomes and health services utilization.

**Figure 3.**
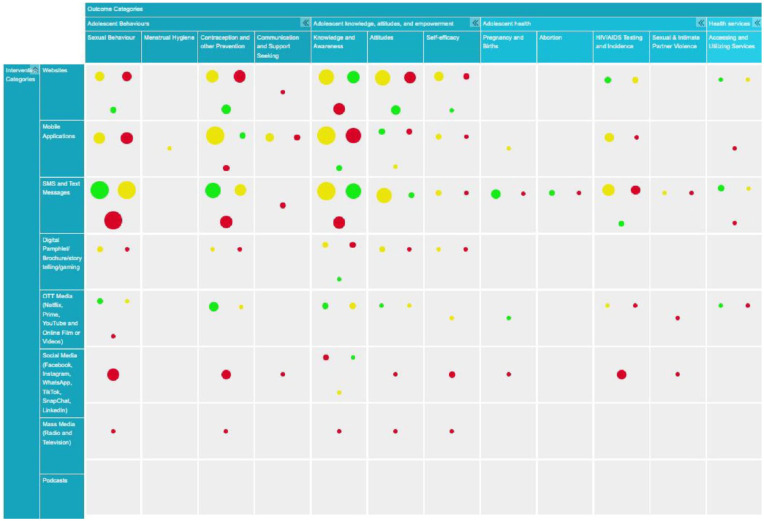
Evidence gap-map.

Areas with less exploration in digital technology include OTT media and social media. There is a moderate amount of research on the influences of OTT media on adolescent behavior (five studies)^9,29,46,47,76^ and knowledge and attitude (four studies),^34,47,57,76^ with limited studies on effects on adolescent SRH outcomes (three studies)^9,34,76^ and healthcare access (two studies).^29,59^ There are also few studies on the impacts of social media on adolescent behavior (four studies),^9,14,27,86^ knowledge and attitude (four studies),^14,36,41,86^ and SRH outcomes (three studies),^9,14,86^ yet none explore its impact on healthcare access and utilization. Moreover, research on the effects of mass media, such as radio and television, on adolescent SRH behavior (one study),^81^ knowledge, attitude and self-efficacy, remains lacking (one study),^81^ with no studies focusing on adolescent SRH outcomes and health service access, indicating an unexplored area. Notably, within the outcome category, the EGM revealed that subcategories like menstrual hygiene (one study),^82^ abortion (three studies),^12,31,52^ and sexual and intimate partner violence (two studies)^9,42^ were less explored (Figure 3 or click here).

## Discussion

To the best of our knowledge, this is the first comprehensive evidence map focused on digital KT tools for adolescent SRH. The findings substantiate the diverse and impactful role of digital KT tools in shaping adolescent SRH knowledge, attitudes, and behaviors, underscoring their significance in promoting informed decision-making and positive SRH outcomes among adolescents. Websites emerge as essential platforms offering accessible and influential avenues for adolescents seeking SRH information and behavior. These websites serve as easily accessible, user-friendly, and potentially influential mediums, catering to adolescents seeking information regarding their sexual and reproductive well-being.^9^ Consistent with prior reviews, these online platforms serve as conduits for disseminating crucial knowledge, shaping attitudes, and potentially modifying behaviors related to SRH.^9,16,17^ Within the evolving digital landscape, mobile apps have emerged as exemplars of innovation and adaptability in addressing adolescent SRH outcomes. These applications present interactive, captivating, and tailored content, serving as an effective means to engage adolescents. These findings align with the literature highlighting the potential of well-designed, evidence-based mobile apps to deliver customized content and interactive features.^6,12^

Moreover, the review underscores the profound influence of SMS/text messages as powerful tools in the domain of adolescent SRH. Supported by extensive literature, these text-based interventions play a significant role in shaping adolescents’ understanding and attitudes toward SRH-related knowledge and behaviors.^9,17,20^ Additionally digital interventions, such as pamphlets, brochures, storytelling, and gaming, exhibit promise in engaging and educating adolescents on SRH matters. Consistent with prior literature, these digital interventions showcase effectiveness in influencing SRH-related knowledge and behaviors among adolescents.^33,34,37^

Our synthesis of the existing EGM highlights the significant gap in understanding the landscape of available digital KT options and their impact on adolescent SRH information. Despite the increasing prevalence of digital platforms, such as social media, OTT media, and technology-driven interventions like radio/TV and podcasts,^87^ their effects on adolescent SRH outcomes remain relatively underexplored. Investigating the influence of these platforms is crucial, as they serve as pervasive sources of information and influence adolescents, shaping their attitudes and behaviors related to SRH.^88^ Furthermore, parental consent may affect the accessibility and effectiveness of these digital KT interventions, particularly on sensitive topics. Some families may have reservations about certain SRH subjects, potentially limiting adolescents’ opportunities to seek and obtain critical information, thereby impacting their informed decision-making.

EGM revealed that the outcomes related to abortion, and sexual and intimate partner violence, were least explored. The lack of work in these areas is a concern for several reasons. First, neglecting outcomes related to abortion, and sexual and intimate partner violence overlooks critical components of comprehensive SRH.^8,14,89^ Second, abortion and intimate partner violence are often stigmatized and surrounded by societal taboo.^8,32,52^ Digital KT tool options have the potential to provide a more discreet and accessible platform for individuals seeking support in these areas. Neglecting these topics may perpetuate stigma and discourage individuals from seeking help.^74,75^ By incorporating these dimensions into the discourse, we can enrich our understanding of how digital interventions can comprehensively address the multifaceted aspects of adolescent SRH, fostering more effective strategies for promoting the ASRH.

The EGM further underscores a concerning scarcity of work on menstrual hygiene within the context of digital interventions for adolescent SRH. This lack of focus on menstrual hygiene is problematic due to the reason that menstrual hygiene is an integral component of SRH and rights,^8,89^ and the omission of KT tools in this area poses a significant gap in our understanding of how digital KT tools can address the unique challenges faced by adolescents in managing menstrual health.

The variation in different numbers of studies in different states in different regions and countries might be due to a high prevalence of SRH issues, the presence of digital technology, and/or the capacity to undertake research in these countries and regions.^90^ There is a need to conduct research and introduce digital technology, potentially in all regions and countries, to understand its impact on ASRH. Key considerations for policy, practice, and research are summarized in Table 1 and discussed subsequently.

**Table 1.** Key policies, practice, and research considerations.

1. Policies should be enacted to encourage and support research initiatives in underrepresented regions (South East and Eastern Mediterranean region) to rectify the current geographical imbalance in understanding the impacts of digital interventions on adolescent health. This would promote a more comprehensive global understanding.
2. Future funding should prioritize the development and implementation of neglected mediums like radio and podcasts, ensuring tailored, culturally sensitive approaches for effective engagement with diverse adolescent populations.
3. We recommend the integration of highly impactful digital mediums such as SMS/text messages, mobile applications, and websites within healthcare systems is necessary to expedite the improvement of adolescent SRH outcomes.
4. The significance of essential areas like menstrual hygiene, abortion, and sexual and intimate partner violence, prioritizing research focused on recognizing and comprehending the influence of digital KT interventions on these crucial aspects should be encouraged.
5. Active promotion and incentivization of international collaborations among researchers and institutions across regions should be undertaken to facilitate knowledge exchange and the sharing of best practices.
6. The sustainability and scaling up of these KT tools in different contexts will not only enhance the impact of digital interventions on adolescent SRH health but also economize research funds. Therefore, there is a critical need to evaluate the scalability of these tools in varied settings to ensure their effective implementation.

The strength of this study is the methodological rigor with which it was performed, which included an extensive search strategy, a comprehensive summary of the results, and an independent assessment of each stage of the study selection process. Moreover, this review provided a holistic, prescriptive model that can be used to scale up the available KT tools in international contexts, and simultaneously leverage significant economies of scale.

## Limitations

Certain limitations of this review warrant acknowledgment. Some articles were excluded for not being written in the English language and no full-text article availability, which may have resulted in studies exploring the impact on a broader range of outcomes being missed. The focus on effectiveness limited our selection to experimental and quasi-experimental studies, omitting cross-sectional, qualitative studies, and gray literature. Further 48% of studies are of moderate-quality evidence. This suggests that findings should be interpreted with caution in light of moderate-quality review evidence.

## Conclusion

The EGM has illuminated the multifaceted landscape of research and knowledge gaps surrounding available digital tools for disseminating knowledge in ASRH. This overview has underscored the geographical disparities in digital tool accessibility, their varying impacts on different facets of SRH, research capacities, and essential study characteristics. Furthermore, the analysis has emphasized the dominance of specific interventions while highlighting persistent research voids in exploring diverse digital platforms and less-represented outcome areas globally. It is imperative that future research endeavors focus on broadening the investigation into various digital platforms and expanding the range of measured outcomes to ensure a more comprehensive understanding and advancement in other critical domains of ASRH. By offering policymakers, healthcare providers, and researchers the ability to gauge evidence accessibility and reliability, EGMs serve as invaluable tools in shaping decisions regarding future research funding.

## Figures and Tables

**Appendix A.**
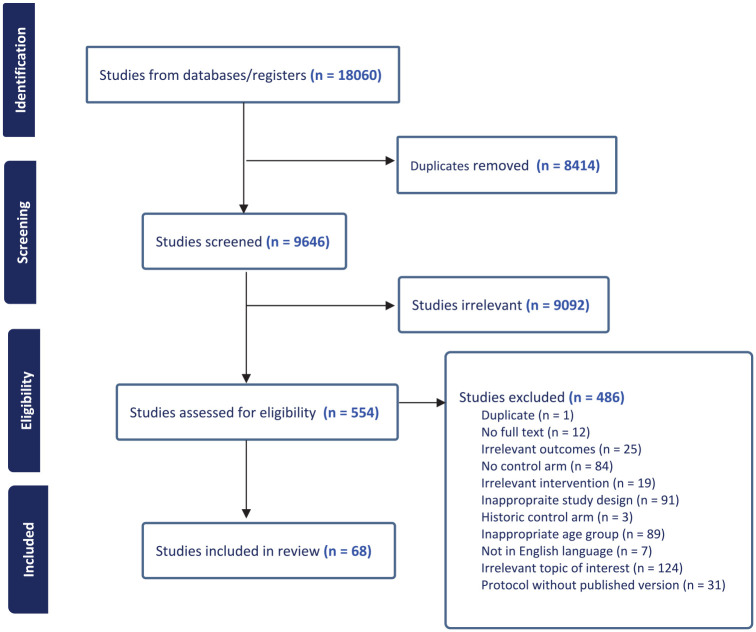
PRISMA flow diagram.

## Appendix

**Appendix B:** Extraction table for primary studies.

Sr. no	Author and year	Country	Study design	Target population (age and sex)	Total participants	Purpose	Intervention (Digital KT tool)	Control group	Outcome	Quality assessment
1	Babalola (2019)^75^	Nigeria	Cluster Randomized control trial	Sex: Female, Age: 18–35 years	Intervention group (*n* = 211), control group (*n* = 344) Total: 565	Assess the effects of exposure to the digital tool on contraceptive ideation and use among women of reproductive age	The Smart Client digital health tool delivered via mobile phone	No intervention	• Considerations for desired family size • Perceive self-efficacy for communicating with a family planning provider • Spousal communication about family size • Spousal communication about contraceptive methods • Misinformation rejection that contraceptives can harm the womb • Current modern contraceptive use	Some concerns
2	Bourdeau (2021)^27^	United States	Randomized control trial	Sex: Male and female with their families Age: 16–17 years	Intervention group (*n* = 206), control group (*n* = 205) Total: 411	Promote healthy decision-making among adolescents and leverage the ongoing impact of parental relationships through older adolescence and young adulthood	Smart Choices 4 Teens, a web-based intervention	No intervention	• Parent-adolescent communication • Birth control • Sex permissive • Health risk • Delaying sex	High risk
3	Brown (2018)^28^	United Kingdom	Factorial design Randomized control trial	Sex: Female and male Age: ⩾16 years	Intervention group 1 (*n* = 2421), group 2 (2357), group 3 (*n* = 2411), control group (*n* = 2396) Total: 8999	Determine whether behaviorally informed SMS primer and reminder messages could increase the return rate of HIV self-sampling kits ordered online	Text message 1. Primer sent 1 day after dispatch plus standard reminders 2. BI reminders (no primer 3. Primer plus BI reminders	Standard reminders sent days 3 and 7 after dispatch (control)	• The primary outcome: The percentage of kits returned according to intervention combination • Secondary outcomes: HIV self-sampling kit return rates by age, sexual behavior and gender identity, ethnicity, and deprivation	Some concerns
4	Chernick (2022)^29^	United States	Pilot Randomized control trial	Sex: Females Age: 14–19 years	Intervention group (*n* = 72), control group (*n* = 74) Total: 146	Assess implementation outcomes and potential efficacy digital health intervention to improve SRH for adolescent females	Multimedia and text messages on patient-centered contraception education	Standard care	• Contraception initiation • Sex in the last 3 months • Consistent condom use • Emergency contraception use • Access the healthcare facility • Intentions toward using birth controls	Low risk
5	Chong (2020)^30^	Colombia	Randomized control trial	Sex: Male and female Age: 14–15 years ninth grade students	Intervention group (*n* = 46 groups), spillover condition (*n* = 46 groups), control group (*n* = 46) Total: 138 groups (4599 participants with an average of 33 students per group)	To see if using information technology in a classroom will assist teenagers navigate the informational challenges associated with sexual education	An internet-based sexual health course covered	No intervention	• Sex knowledge • Attitude: condom use • Sexual activity and condom demand	Some concerns
6	Constant (2014)^31^	South Africa	Randomized control trial	Sex: Female undergoing early medical abortion Age: >18 years (mean age = 25.6 years)	Intervention group (*n* = 234), control group (*n* = 235) Total: 469	Evaluate the effect of automated text messages on anxiety emotional discomfort, and to prepare for symptoms among women undergoing medical abortion	Automated text messages	Standard care	• Experience and acceptability of abortion	Low risk
7	Cordova (2020)^32^	United States	Randomized control trial	Sex: Male and female Age: 13–21 years	Intervention group (*n* = 25), control group (*n* = 25) Total: 50	Effects of storytelling on substance use, sexual risk behaviors, and testing for STIs and HIV	Multi-level mobile health app (through storytelling)	Enhanced usual practice	• Clinician-youth risk communication • Substance use prevention knowledge • Sexual risk prevention knowledge • Sexual risk refusal self-efficacy • STIs and HIV testing	High risk
8	Cortese (2012)^33^	United States	Randomized control trial	Sex: Male and female Age: Not reported (grade 7–10 students)	Total: 151 (sample size for control and intervention group not reported separately)	Evaluate the effectiveness of a web-based tailored intervention in increasing knowledge and encouraging deeper elaborations of health messages related to teen sexual health and decision-making	Web-based tailored educational site on teen sexual health	Nontailored educational Web site on the same topic.	• Sexual health knowledge • Decision making (attitude)	High risk
9	Del Río-González (2021)^34^	Colombia	Randomized control trial	Sex: Male who have sex with men Age: 18–40 years	Intervention group (*n* = 150), control group (*n* = 150) Total: 300	Evaluate the online education-entertainment intervention on HIV knowledge and uptake of HIV testing	Online educational entertainment video	No intervention	• HIV knowledge • HIV testing behavior and intention	Some concerns
10	Dike (2021)^35^	Nigeria	Randomized control trial	Sex: Male and female school children Age: 9 years and above	Intervention group (*n* = 41), control group (*n* = 43) Total: 84	Effect of educational digital storytelling on knowledge and perception of HIV/AIDS among English language speaking children in rural areas of Nigeria	Educational digital storytelling	No intervention	• Knowledge and perception of HIV/AIDS	High risk
11	Ezegbe (2018)^36^	Nigeria	Randomized control trial	Sex: Male and female Age: Junior secondary school children (mean age = 14.63 ± 1.23 years)	Intervention group (*n* = 40), control group (*n* = 40) Total: 80	Determine the efficacy of a rational emotive digital storytelling therapy on knowledge and perception of risk of HIV/AIDS among schoolchildren in Nigeria	Audiovisual intervention at social media digital storytelling (REDStory)	No intervention	• HIV knowledge and perception	Low risk
12	Ujang (2018)^79^	Malaysia	Quasi-experimental	Sex: Male and female Age: 18–19 years	Intervention group (*n* = 96), control group (*n* = 121) Total: 217	Evaluate the effects of mobile phone messaging in improving SRH among late adolescents	Mobile phone text messages (short SMS) covered sexual reproductive health domain	Health talk	• SRH knowledge • HIV/AIDS knowledge and attitude	Some concerns
13	Free (2016)^37^	United Kingdom	Randomized control trial	Sex: Male and female Age: 16–24 years diagnosed with chlamydia or reporting unprotected sex with more than one partner	Intervention group (*n* = 99), control group (*n* = 101) Total: 200	To assess safer sex behaviors in young people by text messages	Automated mobile phone messaging (A theory- and evidence-based safer sex intervention)	The control messages contained checking contact detail but no information regarding sexual health	• Behavioral outcome such as treatment compliance, safe sexual practices, and STI testing	Low risk
14	Gerend (2021)^38^	United States	Randomized control trial	Sex: Male Age: 18–25 years old sexual minority men	Intervention group (*n* = 72), control group (*n* = 76) Total: 148	Efficacy of a text messaging-based HPV vaccination intervention for young sexual minority men	Text messages on sexual health program called txt2protect focused on vaccination, with only a brief mention of other sexual health practices (e.g., condom use and HIV testing)	Control condition messages focused on a variety of sexual health practices with only a brief mention of HPV vaccination	• Primary outcomes: Intervention feasibility, acceptability, and preliminary efficacy (i.e., HPV vaccine initiation) • Secondary outcomes: Attitudes and self-efficacy) • Condom use and HIV testing	Some concerns
15	Gil-Llario (2014)^39^	Spain	Randomized control trail	Sex: Female Age: Age not reported (mean age = 21.3 years old)	Total five intervention groups: Intervention 1: Talk (*n* = 28), Intervention 2: Discussion (*n* = 31), Intervention 3: Role-play (*n* = 31), Intervention 4: Fear (*n* = 33), Intervention 5: Web (*n* = 23), control = 21) Total: 167	Examine the differential effectiveness of six intervention elements on HIV prevention	Informative website: Intervention 1: Informative talk on HIV knowledge Intervention 2: Attitudinal discussion and debate about HIV/AIDS topic Intervention 3: Role-play on risky sexual situations Intervention 4: Fear induction, that is, showing fear-inducing images and videos Intervention 5: Informative website	No intervention	• Knowledge, attitude and self-efficacy • Confidence in condom use • Sexual acts • Perceived fear • Sexual behavior	Some concerns
16	Gilliam (2014)^40^	United States	Randomized control trial	Sex: Female Age: 15–29 years	Intervention group (*n* = 28), control group (*n* = 24) Total: 52	Evaluate the app’s impact on contraceptive knowledge, LARC interest, and selection of a LARC method	Tablet computer programmed with the app on IUD or implants	Standard care	• knowledge of contraceptive • Interest and interest, and selection of a LARC method	Low concern
17	Gonenc (2021)^41^	Turkey	Randomized controlled trial	Sex: Female (midwifery students) Age: Age not reported (mean age = 19.24 ± 1.140 years)	Intervention 1: WhatsApp education group (*n* = 32), Intervention 2: Convention education group (*n* = 35), control group (*n* = 31) Total: 98	Evaluate the effects of sexual education via WhatsApp and the conventional method on the level of sexual knowledge and sexual myths in midwifery students	Intervention 1: Online education via WhatsApp group Intervention 2: Conventional education (face to face)	No intervention	• Sexual health knowledge test, and sexual myths	Some concerns
18	Gonsalves (2019)^42^	Kenya	Randomized controlled trial	Sex: Male and female Age: 18–24 years	Intervention 1: (*n* = 203), Intervention 2: (*n* = 221), control (*n* = 236) Total: 660	Evaluate the effect of the ARMADILLO intervention on SRH-related outcomes	ARMADILLO text messages Intervention 1: One SMS text message Intervention 2: Two pushed messages per week	Usual care	• Primary outcome: Change in myths and misconceptions related to contraception • Secondary outcomes: Change in knowledge, attitudes, and behavior for key SRH outcomes (e.g., knowledge of HIV/AIDS and its transmission, attitudes around violence against women, engagement in sexual activity)	Some concerns
19	Green (2018)^43^	Kenya	Randomized control trial	Sex: Female Age: 15–49 years	Intervention (*n* = 56), control (*n* = 56) Total: 112	Examine the effect of the digital health intervention on contraception uptake	Automated investigational digital health tool (SMS text message) to try the service and complete a free family planning screening (plus bonus)	Message for thanking them for participating in the study only	• Self-reported use of modern contraception	Some concerns
20	Haruna (2018)^44^	Tanzania	Randomized control trial	Sex: Male and female Age: 11–15 years	Intervention 1: Gamified instruction, (*n* = 40), Intervention 2: Gamification (*n* = 40), control (*n* = 40) Total: 120	Investigates the extent to which GBL and gamification could improve the sexual health education of adolescent students	Intervention 1: GBL Intervention 2: Gamification	Traditional teaching (lecture)	• MAKE • Sexual health behavior	Some concerns
21	Haruna (2021)^80^	Tanzania	Quasi-experimental	Sex: Male and female Age: 11–15 years in secondary school	Intervention 1: Gamified instruction (*n* = 40), Intervention 2: Gamification (*n* = 40), control (*n* = 40) Total: 120	Improve sexual health knowledge and understanding of the problems associated with unhealthy sexual practices and address SRH challenges experienced in a low-tech setting.	Intervention 1: Gamified instruction (actual serious games in teaching); Intervention 2: Gamification (making nongame’s, such as game-like learning)	Traditional teaching class	• Motivation, attitude, know-how, and participation in learning	Some concerns
22	Horvath (2017)^45^	United States	Randomized control trial	Sex: Gay and bisexual male Age: 15–24 years	Intervention (*n* = 84), control (*n* = 43) Total: 130	Assessed whether young men who have sex with men’s accept online Get Connected! intervention and impact on subsequent sexual health decision-making	Website-based intervention (online Go connected!)	No intervention	• Intervention system quality scores, sexual health decision-making	Some concerns
23.	Hylton-Kong (2021)^46^	Jamaica	Randomized control trial	Sex: Female Age: 18–25 years	Intervention (*n* = 110), control (*n* = 110) Total: 220	To evaluate video effectiveness on perceived contraceptive safety	LARC-based videos	Intervention to control mosquitoes	• Perceived contraceptive safety	Low risk
24	Lohan (2018)^76^	United Kingdom	Clustered Randomized control trial	Sex: Male and female Age: 14–16 years	Intervention (*n* = 420), control (411) Total: 831	Evaluate an interactive film-based intervention to reduce teenage pregnancy and promote positive sexual health	Interactive film “If I Were Jack”	No intervention	Primary outcome: • Unintended teenage pregnancy rates • Avoidance of unprotected sex via consistent use of contraception Secondary outcomes: • Knowledge, attitudes, skills and intentions relating to avoiding teenage pregnancy	Low risk
25	Jones (2013)^47^	United States	Randomized control trial	Sex: Female Age: 18–29 years	Intervention (*n* = 117), control (*n* = 121) Total: 238	To reduce risk of sexually transmitted HIV in young urban African American women	Video series (soap opera) on love, sex, and choices	HIV prevention text messages	• Changes in unprotected sex (sexual behavior and contraception use) • Knowledge, attitude, and self-efficacy	Some concerns
26	Macharia (2022)^48^	Kenya	Randomized control trial	Sex: Male and female Age: 15–19 years	Intervention (*n* = 146), control (*n* = 154) Total: 300	Investigate the impact of an USSD-based app in increasing adolescents’ knowledge about contraceptives, gender-based stereotypes, STIs, abstinence, and perceived vulnerability, and helping adolescents make informed decisions about their SRH	Mobile phone-based health intervention: USSD-based app	No intervention	Primary outcome: • SRH knowledge and attitude Secondary outcome: • Decision-making on SRH.	High risk
27	Manlove (2021)^49^	United States	Randomized control trial	Sex: Female Age: 18–20 years	Intervention (*n* = 146), control (*n* = 154) Total: 1588	Evaluate pulse, an app-based pregnancy prevention program implemented with women aged 18–20, a population with high rates of unplanned pregnancy	App-based sexual health program (pulse)	General health control app	• Sexual intercourse without using modern contraception • Contraceptive knowledge	Some concerns
28	Marsch (2015)^50^	United States	Randomized control trial	Sex: Male and female Age: 12–18 years	Intervention (*n* = 69), control (*n* = 72) Total: 141	Evaluate changes in knowledge about HIV, hepatitis, and STIs, intentions to engage in safer sex, sex-related risk behavior, self-efficacy to use condoms, and condom use skills	Web-based TES intervention	Traditional education	• Knowledge about HIV, hepatitis, and STIs, intentions to engage in safer sex, sex-related risk behavior, self-efficacy to use condoms, and condom use skills	Some concerns
29	McCarthy (2018)^51^	Tajikistan	Randomised control trial	Sex: Male and female Age: 16–24 years	Intervention (*n* = 275), control (*n* = 298) Total: 537	Assess the effect of the intervention (instant messages) on the acceptability of effective contraceptive methods among young people in Tajikistan	Instant messages about contraception through the app. (access to an app plus intervention messages)	Access to the app plus control messages	• Use of effective contraception, service uptake, knowledge, attitude, perceived norms, personal agency, and intention to use effective contraception	Low risk
30	McCarthy (2019)^52^	Palestine	Randomised control trial	Sex: Female Age: 18–24 years	Intervention (*n* = 229), control (*n* = 235) Total: 464	Assess the impact of mobile text message on attitudes toward the non-permanent effective contraceptive methods available in Palestine	Mobile text message (three messages per day)	Mobile text message (60 messages over 120 days)	Primary outcome: • Acceptability of at least one method of effective contraception Secondary outcome: • Effective contraception use acceptability of individual methods, service uptake, unintended pregnancy, and abortion Other outcomes: Knowledge, attitude, perceived norms, personal agency, and intention	Low risk
31	McRee (2018)^53^	United States	Randomised control trial	Sex: Gay and bisexual men Age: 18–25 years not received HPV vaccine	Intervention (*n* = 68), control (*n* = 73) Total: 150	Pilot test a web-based, Outsmart HPV intervention, to promote HPV vaccination among YGBM	Web-based intervention (individually tailored content called Outsmart HPV intervention)	Standard HPV vaccination information	• HPV vaccination attitudes, beliefs, and intentions	Some concerns
32	Mortimer (2015)^54^	Australia	Randomised control trial	Sex: Male and female Age: 18–29 years	Intervention (*n* = 369), control (*n* = 378) Total: 747	Determine if a web-based PCHMS could increase the uptake of STI screening among a young university population	Web-based Healthy.me (PCHMS)	Redirected to a static webpage informing them of their allocation	• Self-reported being tested for STIs, accessing healthcare facility, attitudes, and intentions toward accessing healthcare services for STI screening	Low risk
33	Musiimenta (2012)^81^	Uganda	Quasi-experimental Pre post intervention	Sex: Male and female Age: 11–16 years	Intervention (*n* = 180), control (*n* = 218) Total: 398	Determine the effect of WSWM intervention on students’ sexual behaviors, HIV/AIDS knowledge, attitudes, and self-efficacy	Computer-based HIV/AIDS Education that includes websites, television, and printouts	No intervention	• HIV/AIDS knowledge, attitudes self-efficacy, sexual behavior, and condom use.	High risk
34	Nalwanga (2021)^82^	Uganda	Quasi-experimental	Sex: Male and female Age: 18–30 years	Intervention (*n* = 556), control (*n* = 556) Total: 1112	Assess the utilization of a MPA to increase access to SRH information, goods, and services among university students in Uganda	MPA	No intervention	• Awareness and utilization of MPA regarding use of contraception, sanitary pads, HIV testing	Some concerns
35	Nielsen (2021)^55^	Sweden	Randomized control trial	Sex: Male and female Age: 18–23 years	Intervention (*n* = 214), control (*n* = 219) Total: 433	Evaluate the effectiveness of a MPA to improve sexual health among youth of Stockholm County in addition to routine care	Smartphone app to promote safe sex among youth called “Skyddslaget” (“protection team”)	A “dumdum” application and routine care	Primary outcome: • Self-reported condom use Secondary outcome: • Self-reported number of partners, occurrence of STIs, pregnancy, and STI tests	Low risk
36	Nik (2018)^83^	Malaysia	Quasi-experimental Pre and post-test design	Sex: Male and female Age: 12 years	Intervention (*n* = 101), control (*n* = 108) Total: 209	Identify the potential of an internet-based program to improve SRH knowledge among Malaysian young people	Online internet-based SRH education	Face to face information	• Adolescents’ knowledge of SRH	Low risk
37	Njuguna (2016)^84^	Kenya	Quasi-experimental	Sex: Female Age: 18–24 years	Intervention (*n* = 300), control (*n* = 300) Total: 600	Determine the effect of an SMS intervention on uptake of HIV testing among female Kenyan college students	SMS text on HIV sensitization messages weekly	Weekly control surveys	• Sexual behavior and HIV testing	Low risk
38	Odeny (2014)^56^	Kenya	Randomized control trial	Sex: Male Age: older than 18 years (74.6% between 18 and 30 years)	Intervention (*n* = 600), control (*n* = 600) Total: 1200	Determine the effect of text messaging to deter resumption of sex before 42 days post circumcision	A series of text messages	Standard care	• Frequency of self-reported resumption of sexual activity	Low risk
39	Ofoegbu (2020)^57^	Nigeria	Randomized control trial	Sex: Male and female Age: Adolescent (mean age = 20.43 ± 0.89 years)	Intervention (*n* = 49), control (*n* = 49) Total: 98	Investigate the impact of an EDSI on HIV risk perception and knowledge among Nigerian adolescents	EDSI	No intervention	• HIV risk perception and knowledge	Low risk
40	Perez-Lu (2022)^58^	Peru	Randomized control trial	Sex: Male and female Age: 13–17 years	Intervention 1 (*n* = 236), Intervention 2 (*n* = 237), control (*n* = 239) Total: 712	To determine whether adolescents able to access SRH information on-demand and via SMS are better able to reject contraception-related myths and misconceptions as compared with adolescents receiving pushed SMS or usual care	Intervention 1: Access to ARMADILLO SMS information on-demand Intervention 2: Access to ARMADILLO SMS information pushed to their phone	No Intervention	• SRH knowledge and contraception-related myths	Some concerns
41	Riese (2019)^59^	United States	Pilot Randomized control trial	Sex: Female Age: 14–16 years	Intervention (*n* = 35), control (*n* = 31) Total: 66	Evaluate the acceptability and feasibility of an electronic sexual health module for inpatient adolescent girls their uptake of sexual health services	Electronic sexual health module included a sexual health assessment, tailored feedback, and a questionnaire to request sexual health services	Electronic sexual health module included a sexual health assessment but no tailored feedback	• Acceptability and feasibility of intervention • Uptake of sexual health services	High concern
42	Rinehart (2020)^60^	United States	Randomized control trial	Sex: Females Age: 13–18 years	Intervention (*n* = 122), control (*n* = 122) Total: 244	Evaluate the feasibility, acceptability, and initial efficacy of a pilot texting intervention (“t4she”) to increase sexual health knowledge and promote protection strategies to reduce unintended pregnancies and STIs	Text messages for Sexual Health Education and Empowerment (t4she)	Standard care	• Knowledge, condom use, and sexual behaviors	Low risk
43	Rokicki (2017)^61^	Ghana	Randomized control trial	Sex: Female Age: 14–24 years	Intervention (*n* = 205), control (*n* = 293) Total: 498	Assess the degree to which mHealth programs reach target adolescents who may be at higher risk of poor SRH outcomes	Text messages about SRH (mHealth intervention)	Placebo message	• Engagement with the mHealth program, SRH knowledge, and self-reported pregnancy	Low risk
44	Sabben (2019)^62^	Kenya	Randomized control trial	Sex: Male and female Age: 15–24 years	Intervention (*n* = 30), control (*n* = 30) Total: 60	To help prevent HIV among young Africans by delaying first sex and increasing condom use at first sex	Narrative-based smartphone game app (Tumaini)	No intervention	• Acceptability of the game • Sexual behavior and knowledge • Communication with parents	Some concerns
45	Schnall (2020)^63^	United States	Randomized control trial	Sex: Male who report same sex attractions (YMSM) Age: 13–18 years	Total: 350	Evaluate the efficacy of MyPEEPS, an evidence-based intervention for reducing reduce sexual risk behaviors and strengthen protective factors among racially and ethnically diverse YMSM	MyPEEPS mobile	Delayed intervention	• HIV prevention self-efficacy, HIV testing, condomless sexual acts, and HIV prevention measures	Some concerns
46	Shegog (2014)^64^	United States	Randomized, two-arm nested design	Sex: Male and female Age: 8th grade students, 12–14 years	Intervention (*n* = 768), control (*n* = 606) Total: 1374	Assess impact of IYG-Tech on psychosocial factors related to sexual behavior-perceptions of friends’ beliefs, reasons for not having sex, condom use self-efficacy, abstinence intentions, negotiating with others to protect personal rules, and improved knowledge about what constitutes healthy relationships	Computer-based sexual health program: t’s Your Game-Tech (IYG-Tech)	State-approved health education usually from a textbook, without any exposure to a structured health education program	• Sexual behavior including and condom use efficacy (1) delayed initiation of specific types of sexual activity; (2) number of lifetime sexual partners; (3) current sexual activity; (4) number of occasions students had sex without a condom; (5) number of partners in the past 3 months; (6) number of partners without a condom in the past 3 months; and (7) use of a condom during last sex	Some concerns
47	Starosta (2016)^65^	United States	Randomized control trial	Sex: Female Age: 17–41 years	Intervention (*n* = 215), control (*n* = 207) Total: 422	To increase condom, use among college women through web-based intervention	Web-based motivational enhancement intervention	Binge drinking	• Intentions to use and attitudes toward condoms	High risk
48	Stephenson (2020)^66^	United Kingdom	Randomized control trial	Sex: Female Age: 15–20 years	Intervention (*n* = 464), control (*n* = 463) Total: 927	To increase the uptake of LARC methods in young women	An interactive website	Standard care	• Knowledge and use of LARC and satisfaction with contraceptive method	Low risk
49	Tan (2022)^67^	Singapore	Pragmatic, randomized controlled trial	Sex: Gay, bisexual, and other men who have sex with men HIV negative (GBMSM) Age: 18–29 years	Intervention (*n* = 150), control (*n* = 150) Total: 300	Evaluate the effectiveness of a popular web drama video series developed by a community-based organization in Singapore for GBMSM on HIV and other STI testing behaviors	Web-based drama video series	Standard care	• Self-reported regular testing and participants’ intentions to test for HIV and chlamydia or gonorrhea	Some concerns
50	Tang (2022)^68^	China	Randomized controlled trial	Sex: Male and female Age: 15–24 years	Intervention (*n* = 50), control (*n* = 46) Total: 96	Evaluate the effect of AIDS fighter health defense on young students in improving AIDS-related knowledge, stigma, and attitude related to high-risk behaviors in Southwest China	AIDS educational mobile game AIDS Fighter Â· Health Defense	AIDS-related knowledge through independent learning on the QQ chat group	• Knowledge awareness rate, stigma score, attitudes of high-risk AIDS behaviors	High risk
51.	Tebb (2021)^77^	United States	Cluster Randomized controlled trial	Sex: Female Age: 14–18 years	Intervention (*n* = 693), control (*n* = 667) Total: 1360	Evaluate the effectiveness of Health-E You/Salud iTu, a mobile health application (app), on knowledge, self-efficacy, and contraception use among Latina adolescents, its impact on visit, quality, and satisfaction	Health-E You/Salud iTu: an mHealth application of patient-centered contraceptive decision-making support	Provided questions to answer about sexual health on iPads	• Knowledge, self-efficacy, and contraceptive use • App satisfaction	Some concerns
52	Turah (2019)^85^	Indonesia	Quasi-experimental pre and post test	Sex: Male and female Age: 15–19 years	Intervention (*n* = 53), control (*n* = 53) Total: 106	Determine the effect of health education using an android application namely Gapin on adolescent knowledge and attitudes about premarital sex	Gapin intervention an android application	No intervention	• Knowledge and attitudes toward premarital sex	Some concerns
53	Widman (2018)^74^	United States	Randomized controlled trial	Sex: Females Age: 14–17 years	Intervention (*n* = 107), control (*n* = 115) Total: 222	Assess the feasibility and acceptability of HEART	HEART, a web-based intervention focused on developing sexual assertiveness skills and enhancing sexual decision-making	Controlled web program focused on cultivating academic growth mindsets	• Self-reported assertiveness, intentions to communicate about sexual health, knowledge regarding HIV and other STDs, safer sex norms and attitudes, and condom self-efficacy	High risk
54	Winskell (2018)^69^	Kenya	Randomised control trail	Sex: Male and female Age: 11–14 years	Intervention (*n* = 30), control (*n* = 30) Total: 60	Pilot test a theory-based, empirically grounded smartphone game for young Kenyans to increase condom use at first sex	Android smartphone game (Tumaini)	No intervention	• Sexual health-related knowledge, self-efficacy, behavioral intention for risk-avoidance strategies, sexual risk communication	Some concerns
55	Wirsiy (2022)^70^	Cameroon	Randomized controlled trail	Sex: Female Age: 10–19 years	Intervention (*n* = 230), control (*n* = 230) Total: 460	Improve perception of adolescent girls on sexo-reproductive health in Cameroon	MASHS	Usual care	• Knowledge, attitude, and practice about sexo-reproductive health	Some concerns
56	Wong (2021)^71^	China	Randomized controlled trail	Sex: Female university students Age: ⩾18 years	Intervention (n=384), control (n=397) Total:781	Development and systematic evaluation of a web-based sexual health literacy intervention called “Smart Girlfriend” for female Chinese university students	Web-based sexual health literacy intervention: The Smart Girlfriend was based on the Dating CAFE initiative	No intervention	• Self-reported consistency of condom use, appraisal of the knowledge, attitudes, norms, and self-efficacy of condom use	Some concerns
57	Ybarra (2021)^72^	Uganda	Randomized controlled trial	Sex: Male and female Age: 18–22 years	Intervention (*n* = 101), control (*n* = 101) Total: 202	Examine the impact of an internet-based HIV prevention program on Information-Motivation-Behavior Skills	A text messaging-based HIV prevention program: InThistoGether (ITG)	Text message focused on general health	Behavior change: Frequency of unprotected sex acts; sustained sexual abstinence, and HIV testing rates	Some concerns
58	Zha (2021)^73^	China	Randomized controlled trail	Sex: Male and female Age: College students >18 years (age not reported)	Intervention (*n* = 56), control (*n* = 55) Total:111	Evaluate a WeChat-based HIV and AIDS educational intervention to enhance knowledge, improve attitudes, and reduce stigma among college students in China	WeChat-based HIV and AIDS educational intervention	No intervention	• HIV- and AIDS-related knowledge, attitudes, and stigma	Some concerns
59	Zhang (2022)^78^	China	Cluster randomized controlled trial	Sex: Female Age: Not reported (mean age = 18.99 years)	Intervention (*n* = 532), control (*n* = 414) Total:946	Investigate the effect of a web-based educational intervention on changing female college students’ willingness and uptake of HPV vaccines and its factors	Web-based health education regarding HPV and HPV vaccines	Health tips unrelated to HPV vaccination or prevention	• Awareness, knowledge, and willingness to be vaccinated against HPV	Low risk

BI, behavioural insights; EDSI, educational digital storytelling intervention; GBL, game-based learning; HEART, Health Education and Relationship Training; HIV, human immunodeficiency virus; HPV, human papillomavirus; LARC, long-acting reversible contraception; MAKE, Motivation, Attitude, Knowledge, and Engagement; MASHS, Mobile-based adolescent sexo-reproductive health scheme; MPA, Mobile phone application; PCHMS, personally controlled health management system; SMS, short message service; SRH, sexual and reproductive health; STD, sexually transmitted disease; STI, sexually transmitted infection; TES, therapeutic education system; USSD, Unstructured Supplementary Service Data; WSWM, World Starts With Me; YGBM, Young Gay and Bisexual Men.

**Appendix C.** Extraction table for review studies.

Sr. no	Author and year	Objective/aim/purpose	Country/setting	Target population	Total number of studies	Study design	KT tool	Intervention	Outcomes	Quality assessment
1	Martin (2020)^9^	To describe existing published studies on online participatory intervention methods used to promote the sexual health of adolescents and young adults	Global	Adolescents and young adults aged between 10 and 24 years	*n* = 60	Systematic review	Facebook, YouTube, MySpace, Twitter, Flickr, Tumblr, Instagram, WeChat	• Information dissemination with participatory components (games, quizzes, discussions) • Online community/discussion • Participation in activities only (including games) • Participatory educational sessions • Personalized assistance	• Sexual health promotion • HIV/sexually transmitted infection prevention specifically • Sexual violence prevention • Hepatitis B virus and hepatitis C virus testing promotion • Improve HIV care linkage • Observe peer influence in sexual situations only	High risk
2	Feroz (2021)^7^	To identify a range of different mHealth solutions that can be used for improving young people SRH in LMICs and highlight facilitators and barriers for adopting mHealth interventions designed to target SRH of young people	LMICs	Young people (adolescents and youth) aged 10–24 years	*n* = 15)	Systematic review	Mobile phones	The most reported use of mHealth was for client education and behavior change communication followed by financial transactions and incentives	• Access to SRH services • Behavior change communication • SRH outcomes • Factors facilitating and impeding uptake of mHealth interventions for young people SRH	High risk
3	Onukwugha (2022)^8^	To describe mHealth intervention components, assesses their effectiveness, acceptability, and cost in improving adolescent’s uptake of SRH services in SSA	SSA	Adolescents aged 10–19 years and young people aged 10–24 years	*n* = 10	Systematic review	• SMS An interactive web-based peer support platform	The interventions focused on shaping knowledge and increasing the use of reproductive health interventions or services. Two studies evaluated SRH knowledge,^29,35^ four assessed contraceptive use/birth control,^29,33,34,39^ three examined pregnancy and fertility intentions.^29,33,34^ One focused on facility childbirth delivery,^33^ two on EBF,^33,34^ four on HIV ART adherence,^31,32,37,38^ and two on sexual behavior^29,34^	• SRH knowledge • Sexual health behavior. • Contraceptive/birth control access and use • ART adherence	Low risk
4	Guse (2012)^14^	To summarize the currently published evidence-based on the effectiveness of new digital media-based sexual health interventions for adolescents aged 13–24 years	Global	Adolescents aged 13–24 years	*n* = 10	Systematic review	Internet	The shortest program was a single e-mail and the longest consisted of 24 45-min sessions administered over 2 years. Two of the interventions were delivered to adolescents in rural settings. Two of the interventions enrolled HIV-positive youth, and one enrolled youth with substance use disorders	• Youth behaviors, including initiation of vaginal sex recent sexual intercourse, frequency of sex, number of sexual partners, condom use, and sex while under the influence of drugs or alcohol • Adherence to medication. among young HIV-positive participants and alterations to public profiles on an SNS • Attitudes, self-efficacy, and intentions regarding sexual abstinence • Knowledge-based outcomes pertaining to HIV/ STIs, condoms, pregnancy, and emergency contraception	High risk
5	Saragih (2021)^16^	To explore the meta-effects of telehealth interventions on self-efficacy of using condoms, condom use practices, and sexually transmitted infection testing behaviors among adolescents	Global	Adolescent population aged 13–24 years old	*n* = 15	Systematic review and meta-analysis of randomized controlled studies	Web-based and game-based interventions	The telehealth interventions were designed to influence knowledge, attitudes, norms, self-efficacy, and behaviors; a range of sexual health and general health topics were addressed, including substance use, safer sex strategies, sexual risk behaviors, and risk-reducing behaviors including condom use, STI testing practices, and contraceptive options. A variety of professionals delivered the telehealth interventions, including clinicians (*n* = 3), a school nurse (*n* = 1), researchers in tropical medicine (*n* = 2 studies), a public health researcher (*n* = 1 study), a social worker (*n* = 1 study), and psychologists (*n* = 4 studies). The frequency of intervention delivery was most often weekly, with each session lasting 20–45 min, and the sessions occurring over a 3-week to 6-month period of time	• Knowledge, attitude, self-efficacy for condom use, and sexual behavior • Being screened/tested for sexual transmitted infections	Low risk
6	Nwaozuru (2021)^17^	To summarize what is known, and what we need to know about implementing mhealth interventions for HIV/STI prevention targeting young people in LMICs	LMICs	Young people aged 10–24 years	*n* = 11	Systematic review and meta-analyses	Mobile phones, SMS	The mHealth components across the six interventions were delivered using three modalities: (1) as mobile applications, (2) as phone-based SMSs, and (3) as web-based application. Specifically, two interventions used mobile phone applications to provide HIV/STI prevention services and information. One of the interventions was delivered as a narrative-based game for android smartphones^33^ and one used WeChat—a messaging mobile application	• Acceptability, appropriateness, and feasibility • Condom use and sexual and reproductive health knowledge. • Sexual intercourse after male circumcision	High risk
7	Handschuh (2019)^20^	To synthesize research examining the association between adolescent sexing and sexual activity	Global	Adolescents 10–19 years of age	*n* = 6	Systematic review and meta-analysis	Mobile phones, SMS	Sexting as sending either a written message or an image, one differentiated between sending a message and sending an image	• Sexual behavior	Some concerns
8	Jones (2014)^86^	To examine the effectiveness of social media and text messaging interventions designed to increase STD knowledge, increase screening/testing, decrease risky sexual behaviors, and reduce the incidence of STDs among young adults aged 15 through 24 years	Global	Young adults 15–24 years	*n* = 11	Systematic review	SMS messaging and e-mail communications	Intervention modes included SMS messaging via text, e-mail communications, and internet-based health education programming	• *STD knowledge*: Significant increases in STD knowledge, including increased understanding of sexual protection methods and transmission • STD screening/testing • Increases in STD testing among participants after the intervention • *Sexual risk behavior*: Sexual risk behaviors were examined in 10 studies • Self-efficacy/intention • Significant increases in condom use self-efficacy and intention	High risk
9	L’Engle (2016)^12^	To assess strategies, findings, and quality of evidence on using mobile phones to improve ASRH by using the mERA checklist recently published by the World Health Organization mHealth Technical Evidence Review	Global	Adolescents aged 10–24 years	*n* = 35	Systematic review	Mobile phones	mHealth intervention programs where mobile phones were used to address ASRH	• Knowledge and STI testing • Increased sexual health knowledge and awareness, lower rates of unprotected sex and higher rates of condom use, and greater STI testing • Adolescents commonly asked about sexual acts and practices, physical and sexual development, abortion, and contraception and unplanned pregnancy. *Confidentiality*: Adolescents y liked the confidentiality of mobile phone communication and found the SRH content simple to understand, informative, and easily shared • *HPV vaccine*: Increased HPV vaccination through vaccination reminders sent via SMS to parents or teens	High-risk

ART, antiretroviral therapy; ASRH, adolescent sexual and reproductive health; EBF, exclusive breastfeeding; HPV, human papillomavirus; LMIC, low- and middle-income country; mERA, mHealth Evidence Reporting and Assessment; SMS, short message service; SNS, Social Networking Site; SRH, sexual and reproductive health; SSA, Sub-Saharan Africa; STD, sexually transmitted disease; STI, sexually transmitted infection.

## Declarations

**Ethics approval and consent to participate:** Not applicable.

**Consent for publication:** Not applicable.

**Availability of data and materials:** Not applicable.
